# A Review of the Hereditary Component of Triple Negative Breast Cancer: High- and Moderate-Penetrance Breast Cancer Genes, Low-Penetrance Loci, and the Role of Nontraditional Genetic Elements

**DOI:** 10.1155/2019/4382606

**Published:** 2019-07-09

**Authors:** Darrell L. Ellsworth, Clesson E. Turner, Rachel E. Ellsworth

**Affiliations:** ^1^E-Squared Genomic Solutions, Johnstown, PA, USA; ^2^Murtha Cancer Center/Research Program, Uniformed Services University and Walter Reed National Military Medical Center, Bethesda, MD, USA; ^3^Henry M Jackson Foundation for the Advancement of Military Medicine, Bethesda, MD, USA

## Abstract

Triple negative breast cancer (TNBC), representing 10-15% of breast tumors diagnosed each year, is a clinically defined subtype of breast cancer associated with poor prognosis. The higher incidence of TNBC in certain populations such as young women and/or women of African ancestry and a unique pathological phenotype shared between TNBC and BRCA1-deficient tumors suggest that TNBC may be inherited through germline mutations. In this article, we describe genes and genetic elements, beyond* BRCA1* and* BRCA2*, which have been associated with increased risk of TNBC. Multigene panel testing has identified high- and moderate-penetrance cancer predisposition genes associated with increased risk for TNBC. Development of large-scale genome-wide SNP assays coupled with genome-wide association studies (GWAS) has led to the discovery of low-penetrance TNBC-associated loci. Next-generation sequencing has identified variants in noncoding RNAs, viral integration sites, and genes in underexplored regions of the human genome that may contribute to the genetic underpinnings of TNBC. Advances in our understanding of the genetics of TNBC are driving improvements in risk assessment and patient management.

## 1. Introduction

Breast cancer is a complex disease characterized by clinical, pathological, and molecular heterogeneity, which may influence risk assessment, diagnosis, treatment, and clinical outcomes [1]. Pathological characterization of breast disease includes a number of variables such as histological architecture, degree of cellular differentiation, tumor size, presence of local or distant metastasis, and hormone receptor and human epidermal growth factor receptor 2 (HER2) status.

Triple negative breast cancers (TNBC), which do not express the estrogen (ER) or progesterone receptors (PR) and have little or no HER2 protein expression, account for 10-15% of breast cancers diagnosed each year [2]. TNBC represents an aggressive form of disease, often diagnosed at a later stage, characterized by high-tumor grade, larger size, poorly differentiated histology, more frequent lymph node metastases, and younger age at diagnosis [3]. TNBC is more likely to present as an interval cancer, appearing between screening mammograms, possibly due to higher proliferation rates than other tumor types [4]. Risk of distant metastasis and death are significantly higher in patients with TNBC within five years of diagnosis [3], and TNBC displays distinctive patterns of metastasis with a higher affinity for lung, brain, and distant lymph nodes compared to other subtypes.

Like all types of breast cancer, TNBC exhibits marked heterogeneity in terms of histology, patterns of metastatic dissemination, response to therapies, and patient outcomes. While the majority of TNBC are invasive ductal carcinomas, other histologies may be triple negative as well, with five-year survival outcomes ranging from 100% in patients with medullary tumors to 56% in those with metaplastic TNBC [5]. Although there is significant overlap between TNBC and basal-like tumors, as defined by immunohistochemistry (IHC) and patterns of gene expression, 28% of TNBC are classified as luminal A, luminal B, HER2-enriched, or normal-like [6]. Evaluation of TNBC at the gene expression level has shown variability in levels of estrogen related genes, genes involved in oxidation reduction, and proliferation genes, suggesting that additional subclassification of TNBC is warranted [7]. Cluster analysis of 587 TNBC identified six subtypes including basal-like 1, basal-like 2, immunomodulatory, mesenchymal, mesenchymal stem-like, and luminal androgen receptor, each of which may be responsive to different targeted or chemotherapeutic agents [8].

A number of risk factors have been associated with TNBC that have not been linked to increased risk for other cancer subtypes. In contrast to luminal A tumors, TNBC/basal-like tends to be associated with younger age at diagnosis, African ancestry, younger age at menarche and at first full-term pregnancy, higher parity, lack of breastfeeding, and higher BMI and waist-to-hip circumference ratio [2, 9–11]. The frequency of TNBC in African Americans (29.8%) is intermediate between that in West African women (53.2%) and White American women (15.5%), suggesting a genetic component to TNBC [12]. Strong associations between TNBC and BRCA mutation status have been reported: 70-90% and 16-23% of breast tumors in* BRCA1* and* BRCA2 *mutation carriers are TNBC [11]; however, germline mutations in* BRCA1* and* BRCA2* only account for 15.4% of patients with TNBC [13], and the prevalence of* BRCA1* and* BRCA2 *mutations is lower in African American women (20.4%) with TNBC compared to European American women (33.3%) [14]. These data suggest that TNBC has a genetic component and genes other than* BRCA1* and* BRCA2* may play a role in disease etiology. In this review we examine current data regarding the contribution of germline mutations in high- and moderate-penetrance genes to TNBC. In addition, we evaluate the latest genome-wide association studies (GWAS) and candidate gene approaches to identify low-penetrance genes. Finally, we consider the role of nontraditional genetic variants including single nucleotide polymorphisms (SNPs) in microRNA (miRNA) binding sites, retroelements, and novel sequences not present in the current reference genome in the etiology of TNBC.

## 2. Methods

Relevant literature was identified by searching the PubMed database (https://www.ncbi.nlm.nih.gov/pubmed). Search terms included TRIPLE NEGATIVE BREAST CANCER, GENETICS, HEREDITARY CANCER, miRNA, and VIRUS INTEGRATION. Only articles written in English were included. To ensure data presented here were current, articles published within the last 12 months and/or meta-analyses are highlighted.

## 3. GENES

### 3.1. High-Penetrance Breast Cancer Genes

#### 3.1.1. BRCA1 and BRCA2

*BRCA1* and* BRCA2* are known to be tumor suppressor genes that function in DNA repair pathways. Cells lacking functional BRCA1 or BRCA2 are deficient for double-stranded break repair, resulting in genomic instability that leads to cancer predisposition. Current clinical data suggest BRCA1- and BRCA2-deficient tumors may have heightened sensitivity to platinum agents or poly (ADP-ribose) polymerase I (PARP) inhibitors [25]. In a large collection of families with hereditary breast cancer (n=237), 52% of families had disease that was likely attributable to mutations in* BRCA1* while 32% had disease linked to* BRCA2* [26]. Rebbeck et al. investigated whether the location or type of* BRCA1/BRCA2* mutations is associated with variation in breast and ovarian cancer risk. Patients carrying mutations in exon 11 of* BRCA1* appeared to have different disease phenotypes than patients carrying other* BRCA1* mutations. Similarly, mutations in exon 11 of* BRCA2* were associated with variability in breast and ovarian cancer risk [27]. Mutations in both genes have been associated with increased risk of TNBC albeit at different frequencies and within different age groups.

The first breast cancer susceptibility gene,* BRCA1*, was identified in 1994 [28].* BRCA1* is located on chromosome 17q21 and is comprised of 24 exons, 22 of which encode an 1863 amino acid protein. The BRCA1 protein has multiple sequence motifs including RING, DNA-binding, and BRCA1 C-terminus (BCTR) domains that allow BRCA1 to interact with other proteins and assist in subcellular localization.* BRCA1* is a tumor suppressor gene that contributes to repair of damaged replication forks and double-strand breaks, transcriptional regulation in response to DNA damage, chromatin remodeling, and regulation of cell division, apoptosis, and transcription [29].* BRCA2* is a tumor suppressor gene located on chromosome 13q12 that was identified in 1995 [30].* BRCA2* has 27 exons and the BRCA2 protein interacts with RAD51 through the BRC motif. BRCA2 is also a transcriptional coregulator involved in DNA repair through homologous recombination.

As early as 1998, histological characterization revealed that in comparison with sporadic tumors, tumors in* BRCA1* mutation carriers exhibited a distinct phenotype that includes high mitotic counts, pushing margins, and lymphocytic infiltration [31]. Histologic characteristics of TNBC also include high-grade with high mitotic indices, regions of central necrosis, conspicuous lymphocytic infiltrate, and pushing borders [32]. In fact, TNBC represents the predominant tumor type in patients with* BRCA1* mutations, accounting for 71% (range 42-100%) of tumors, while TNBC has been diagnosed in only 25% of patients with germline* BRCA2* mutations [33]. In contrast, the frequency of* BRCA1* or* BRCA2* mutations in women with TNBC is generally lower, with an average of 35% (range 9-100%) and 8% (2-12%) of women with TNBC harboring germline BRCA1 and BRCA2 mutations, respectively. In addition to differences in mutation frequency, age distribution differs between* BRCA1* and* BRCA2 *positive patients with TNBC, with an average age at diagnosis of 47.2 years and 58.8 years in those with* BRCA1* and* BRCA2* mutations, respectively [34]. In a recent study that included over 10,000 patients with TNBC, germline mutations were associated with odds ratios of 16.27-26.90 for* BRCA1* and 5.42-6.33 for* BRCA2* [35].

#### 3.1.2. PALB2

**Figure 1:**
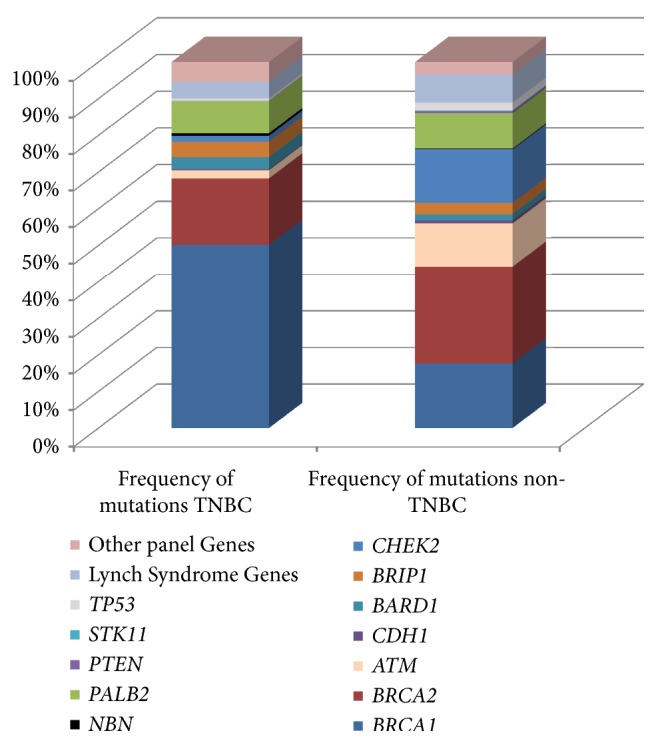
Frequency of mutations by gene within women carrying pathogenic germline mutations with TNBC (n=692) or non-TNBC subtypes (n=2,696). Adapted from Buys et al. 2017.

The BRCA2 binding protein known as partner and localizer of BRCA2 (*PALB2*) stabilizes and regulates BRCA2 through localization and stabilization within important nuclear structures such as chromatin and the nuclear matrix and by promotion of recombination repair and checkpoint functions [36].* PALB2* was recently classified as a high-risk breast cancer gene with an odds ratio (OR) of 7.46 [95% confidence interval (CI)=5.12-11.19] [37]. Mutations in* PALB2* are associated with aggressive disease. Over half (54.5%) of the familial and sporadic breast cancer patients from Finland who carried the 1592delT* PALB2* mutation presented with TNBC compared to other familial (12.2%) or sporadic (9.4%) breast cancer patients [38]. Similarly, in a predominantly European-Caucasian cohort of women, 46% of tumors in women with* PALB2* mutations were TNBC [39]. When mutation profiles were determined in a cohort of 4,797 women diagnosed with TNBC, panel testing performed at Myriad Genetics, found that 1.3% of women had pathogenic variants in* PALB2* (Figure 1) [40]. In a similar cohort of 1,824 women with TNBC unselected for family history of cancer, deleterious mutations in* PALB2* were detected in 1.2% of patients [41]. In a group of 347 Australian women with TNBC the prevalence of deleterious* PALB2* germline mutations was ~1% [42]. A recent study of 10,901 women with TNBC (8,753 women with clinical test results and 2,148 tested in the research setting) found that germline mutations in* PALB2 *were associated with high-risk of TNBC (OR=14.41; 95%CI=9.27-22.60) and were enriched in patients with TNBC compared to non-TNBC tumors (OR2.12; 95% CI=1.63-2.74) [35].

#### 3.1.3. TP53

Tumor protein p53 (*TP53*), which encodes the p53 phosphoprotein, is a tumor suppressor gene that plays a critical role in each of the 10 Hallmarks of Cancer as defined by Hanahan and Weinberg [43]. As a result of this functional diversity, the p53 signaling pathway is at least partially disrupted in most human cancers and* TP53* mutations are the most frequent genetic changes seen in human cancers [44]. Although the frequency of somatic mutations in* TP53* is higher in basal-like tumors than any other subtype [45], germline mutations in* TP53* have not been associated with an increased risk of TNBC. The mutation rate for* TP53* in a cohort of 2,134 BRCA1/BRCA2 mutation negative women with familial breast cancer was 0.52% and* TP53* mutations carriers showed enrichment for HER2+ tumors [39]. In a large cohort of 35,409 women with a single diagnosis of breast cancer, mutations in* TP53* were detected in 0.7% of women with TNBC compared to 2.1% of those with non-TNBC subtypes [40]. In 133 women from Taiwan with early-onset and/or family history of breast cancer, only two women carried a pathogenic mutation in* TP53* and both had ER+/HER2+ tumors [46]. Similarly, only one of 1,824 women with TNBC evaluated by Couch et al. [41] carried a* TP53* mutation. These results suggest that germline mutations in* TP53* are not associated with increased risk of TNBC.

#### 3.1.4. PTEN

Phosphatase and tensin homolog (*PTEN*) is a tumor suppressor gene involved in the regulation of the phosphoinositol-3-kinase and AKT signaling pathways and control of cellular proliferation and survival.* PTEN* is the second most frequently mutated gene in human cancers (after* TP53*) and germline mutations in* PTEN* are frequently observed in cancer susceptibility syndromes [47]. PTEN has recently been shown to protect the genome from instability by maintaining chromosomal integrity. While women with Cowden syndrome who carry germline mutations in* PTEN* have a lifetime risk of breast cancer of 50% [48], there is no consistent breast cancer phenotype associated with* PTEN* mutations. Most* PTEN*-associated tumors are more likely to be luminal than TNBC. Observations that (1) prevalence of pathogenic mutations in* PTEN* did not differ significantly in women with TNBC (n=692) compared to those with non-TNBC tumors (n=2,696) [40] and (2) only one deleterious mutation in 267 patients was observed [41] support the idea that mutations in* PTEN* are not associated with increased risk of TNBC.

#### 3.1.5. STK11

The serine/threonine protein kinase 11 (*STK11*) gene is a highly penetrant breast cancer gene that regulates energy metabolism and cell polarity. Patients who carry mutation in* STK11* present with Peutz-Jeghers syndrome with high risk for various cancers, including breast (lifetime risk 24-54%) and cervical cancers [49]. Currently, there is little evidence to support an association between germline mutations in* STK11* and TNBC as mutations in* STK11* were not observed in (1) a cohort of 2,134 BRCA1/BRCA2 mutation negative women with familial breast cancer, (2) 1,824 women of primarily white ethnicity with TNBC [39, 41], and (3) 4,797 women of mixed ethnicities with TNBC [40].

#### 3.1.6. CDH1

The Cadherin 1 (*CDH1*) gene encodes an adhesion molecule involved in maintenance of epithelial cell morphology. Germline mutations in* CDH1* have been associated with increased risk of Hereditary Diffuse Gastric Cancer, a cancer predisposition syndrome associated with increased lifetime risk of breast cancer, particularly invasive lobular carcinoma (ILCA) [48]. Given that ILCAs are frequently ER+, an association between germline mutations in* CDH1* and TNBC is unlikely. Accordingly, germline mutations in* CDH1* were rare (0.0-0.3%) in women with TNBC [40, 41].

### 3.2. Moderate-Penetrance Breast Cancer Genes

#### 3.2.1. RAD51D

The human RAD51, S Cerevisiae, homolog of D (*RAD51D*) gene plays an important role in maintaining genomic integrity through homologous recombination and repair of double-stranded breaks and inter-strand cross-links in DNA. Mutations in* RAD51D* are associated with a >3-fold increased risk of breast cancer. Mutation rates in patients with TNBC range from 0.20 to 0.95% and tend to be higher in women with TNBC (0.90%) compared to those with non-TNBC tumors (0.5%) [40, 41, 50, 51]. Recent data from Shimelis et al. [35] found that although the mutation frequency of* RAD51D* in 8,243 patients with TNBC was low (0.3%), risk of developing TNBC was high (OR 6.97; 95% CI =2.6-18.66). Together, these data suggest that although the frequency of mutations in* RAD51D* is low, mutation carriers are at increased risk for TNBC.

#### 3.2.2. ATM

The ataxia telangiectasia mutated (*ATM*) gene encodes a phosphatidylinositol 3-kinase thatphosphorylates key substrates involved in DNA repair and control of the cell cycle. In a large cohort of European women (42,671 cases and 42,164 controls) an association with overall breast cancer risk was observed with the c.7271 T>G mutation; however, as tumors were not stratified by subtype, a specific link to TNBC could not be determined [52]. In a group of Polish women with TNBC unselected for family history, one woman out of 158 with TNBC harbored a mutation in* ATM* whereas no* ATM* mutations were detected in 44 women with non-TNBC hereditary breast cancer [53]. Additional studies observed an enrichment of* ATM* mutations in patients with ER positive tumors [39, 46, 54] and a five-fold increase in* ATM* mutations in patients with non-TNBC compared to TNBC tumors [40].

#### 3.2.3. CDKN2A

The cyclin-dependent kinase inhibitor 2A (*CDKN2A*) gene is a tumor suppressor gene involved in cell cycle regulation. [37]. The role of germline mutations in* CDKN2A* in hereditary breast cancers has been difficult to study due to the limited number of variants observed in case-control studies. A mutation frequency of 1.2% has been reported in 692 patients with TNBC compared to 0.9% in 2,696 patients with non-TNBC [40].

#### 3.2.4. MSH2

The MutS, E. coli, homolog of 2 (*MSH2*) gene is involved in DNA mismatch repair and is associated with autosomal dominant Lynch Syndrome. Mutations in* MSH2* may contribute to genomic instability and an increased mutation rate in cancer cells. Evaluation of the G322D variant of* MSH2* in 70 Polish women with TNBC and age-matched controls revealed that the D allele was associated with decreased risk of TNBC (OR=0.11; 95 % CI=0.05-0.21) [55]. A relatively low mutation rate (0.7%) was observed in* MSH2* in women with TNBC compared to 1.2% in women with non-TNBC [40].

#### 3.2.5. CHEK2

Checkpoint kinase 2 (*CHEK2*) encodes a serine threonine kinase involved in DNA repair that serves as a cell cycle checkpoint regulator and tumor suppressor gene. Mutations in* CHEK2* have been associated with various forms of cancer. A large study evaluating* CHEK2* mutations in breast cancer patients from Poland found that* CHEK2* carriers were significantly more likely to have ER+ (OR = 3.9; 95% CI = 2.7–5.4) than ER- (OR = 2.1; 95% CI = 1.3–3.3) tumors [56]. In a similar study,* CHEK2* mutations were enriched in Polish women with hereditary non-TNBC (11.3%) compared to those with TNBC (1.3%) [53]. In 35,409 women subjected to panel testing, the frequency of pathogenic* CHEK2 *mutations was 1.6% in women with TNBC compared to 14.3% in those with other phenotypes [40].

#### 3.2.6. BARD1

The BRCA1-associated RING domain 1 (*BARD1*) gene encodes a protein that interacts with BRCA1 to form a heterodimer, which functions in DNA repair. The heterodimer, essential for BRCA1 stability, may be disrupted by tumorigenic mutations in* BARD1* in patients with breast or ovarian cancer. Of 42 women with TNBC enrolled in the neoadjuvant Trial of Principle study, four harbored missense or nonsense mutations in* BARD1*, of which two (1347A>G and Arg658Cys) have been confirmed as pathogenic [57]. In a study of 105 women with TNBC from Spain,* BARD1* mutations were detected in two patients (1.9%) [50]. Likewise, nine (0.5%) of the 1,824 women in the Triple Negative Breast Cancer Consortium (TNBCC), had* BARD1* mutations [41]. Although not exclusive to TNBC, the mutation frequency in* BARD1* was 3.3% in women with TNBC compared to 1.7% in women with non-TNBC [40]. In a study of 4,032 Caucasian women with TNBC, the mutation rate of* BARD1* was 0.7% compared to 0.2% in women with non-TNBC and the OR for an association with TNBC compared to non-TNBC disease was 3.73 (95% CI=2.3-5.95) [35].

### 3.3. Low-Penetrance Breast Cancer Loci

Mutations in high- and moderate-penetrance breast cancer genes account for ~14% of all TNBC cases [40, 41]. Genome-wide association studies (GWAS) over the last decade have identified SNPs that are associated with breast cancer risk in an additive fashion. In an early study to identify susceptibility loci for breast cancer, ~266,000 SNPs across the genome were genotyped in 408 breast cancer cases with a strong family history and 400 controls from the United Kingdom. In the second phase of this study, ~12,000 SNPs that showed an association with breast cancer in phase I were genotyped in an additional 3,990 cases and 3,916 controls [58]. To determine whether any SNPs were reliably associated with breast cancer risk, the 30 most significant SNPs from phase II were further validated in an additional 21,860 cases and 22,578 controls. Six SNPs were associated with increased risk (P<10^−5^), including SNPs in or near the fibroblast growth factor receptor 2 (*FGFR2*; rs2981582) gene, lymphocyte-specific protein (*LSP1*; rs3817198), mitogen-activated protein kinase kinase kinase 1 (*MAP3K1*; rs889312), and tox high mobility group box family member 3 (*TOX3*; rs12443621 and rs8051542) and in the chromosome 8q24 region (rs13281615). These gene regions were further investigated by ER status in 23,039 cases and 26,273 controls from the Breast Cancer Association Consortium (BCAC) [59]. SNPs rs2981582 in* FGFR2* and rs13281615 (in 8q24) were more strongly associated with ER+ than ER- disease. Although rs3803662 showed the strongest association with ER- tumors, with women homozygous for the variant allele (AA) having an OR of 1.28 (95% CI=1.13-1.45), risk was higher for women with ER+ disease (OR=1.48, 95% CI=1.37-1.60). A 2011 study evaluated breast cancer risk associations with eight SNPs identified through GWAS and two in the candidate genes caspase 8, apoptosis-related cysteine protease (*CASP8*), and transforming growth factor, beta-1 (*TGFβ1*) by immunohistochemistry-defined subtypes [18]. Within the 885-1,644 TNBC cases available for study, five SNPs were significantly associated (P<0.02) with TNBC including rs3803662 (*TOX3*; OR=1.21; 95% CI=1.11-1.30), rs889312 (*MAP3K1*, OR=1.11; 95% CI=1.02-1.20), rs3817198 (*LSP1*, OR=1.11; 95% CI=1.03-1.21), rs13387042 (chromosome 2q35, OR=1.12; 95% CI=1.05-1.21), and rs1982073 (*TGFβ1*, OR=1.11; 95% CI=1.01-1.23). In a meta-analysis of 4,754 ER- breast cancer cases and 31,663 controls from three GWAS, SNP rs2284378 on chromosome 20q11 was associated with ER- tumors (P=1.1x10^−8^) and showed a stronger association with TNBC (OR=1.16, P=6.4x10^−3^) than for ER-/HER2+ tumors (OR=1.07; P=0.41), although the differences did not reach statistical significance [60]. In a second meta-analysis of three GWAS including 4,193 ER- breast cancer cases and 35,194 controls, combined with 40 follow-up studies, variants at rs4245739 located in the 3′ region of the mouse double minute 4 homolog (*MDM4*) oncogene on chromosome 1q32.1 seemed to be specific to TNBC [16].

In addition to the loci summarized above, a GWAS approach identified the 19p13 chromosomal region as a modifier of breast cancer risk in* BRCA1* mutation positive individuals [23]. Five SNPs from 19p13 were genotyped in 2,301 women with TNBC and 3,949 controls to evaluate the association between the 19p13 locus and TNBC in the general population. Minor alleles for SNPs rs8170 (OR per A allele =1.28, 95% CI =1.16-1.41) and rs2363956 (OR per C allele=0.80, 95% CI 0.74-0.87) were associated with TNBC risk in women without* BRCA1* mutations. In the TNBCC, 22 known breast cancer susceptibility loci were studied in 2,980 Caucasian women and 4,978 controls to assess relationships with TNBC. Two SNPs from the 19p13.1 locus [rs8170 (P=2.25x10^−8^) and rs8100241 (P=8.66x10^−7^)] were associated with risk of TNBC, as were SNPs from the estrogen receptor (*ESR1*; rs2046210 and rs12662670),* RAD51L1* (rs999737), and* TOX3* (rs3803662) [21]. Subsequent studies in the BCAC using 48,869 breast cancer cases and 49,787 controls demonstrated that rs8170 was a TNBC-specific risk variant (OR=1.25; 95% CI=1.18-1.33) [24]. A haplotype analysis in this study that included both rs8170 and rs8100241 found that the C-G and T-G haplotypes were both associated with risk of TNBC (C-G OR=1.17; 95% CI=1.09–1.25 and T-G OR=1.35; 95% CI=1.25–1.46) compared with the C-A haplotype.

A GWAS that included women of both European ancestry (1,718 ER- cases, 3,670 controls) and African ancestry (1,004 ER- cases, 2,745 controls) identified a SNP on chromosome 5p15 (rs10069690) from the telomerase reverse transcriptase (*TERT*) – cleft lip and palate-associated transmembrane protein 1-like (*CLPTM1L*) gene region that was associated with TNBC. Combining genotype data from multiple studies for rs10069690 produced a per allele OR of 1.25 (95% CI=1.16-1.34, P=1.1x10^−9^) for risk of TNBC. For women with TNBC diagnosed at <50 years of age, the risk increased to 1.48 (95% CI=1.30-1.68, P=1.9x10^−9^) [61]. Lack of an association with ER+/HER2+ or ER+/HER2- disease suggests that, as observed for the chromosome 19p13 locus, the* TERT-CLPTM1L* locus is specific to TNBC. In a subsequent validation analysis using 15,252* BRCA1* and 8,211* BRCA2* mutation positive individuals to assess disease subtype-specific associations for 74 previously identified breast cancer susceptibility loci, several chromosomal regions discussed above, including 5p15.33 (*TERT*), 6q25.1 (*ESR1*), and 19p13.11, showed a significant association with increased risk of TNBC in* BRCA1* mutation positive individuals [20].

Pooled analysis of the Collaborative Oncological Gene-Environment Study (COGS) and TNBCC SNP data further refined the GWAS data [17]. Multiple data sets, consisting of 22 studies from 7 different countries were combined in a two-stage analysis. Evaluation of SNPs from 3,677 women with TNBC and 4,708 controls supported the association of 25 known breast cancer susceptibility loci, including 2q35,* LGR6*,* MDM4*,* TERT*,* ESR1*,* TOX3*, and 19p13.1 with TNBC. Newly identified associations with TNBC were observed for an additional 15 SNPs from 14 loci. Interestingly, SNPs in* CASP8*,* MAP3K1*,* LSP1*, and* FTO* were not found to be associated with risk of TNBC. More recently, Milne et al. performed GWAS in 21,468 patients with ER- disease and 18,908 BRCA1 mutation positive individuals combined with 100,594 controls [15]. When evaluating the subset of individuals with TNBC, associations with 10 previously reported loci were replicated and 10 new susceptibility loci were identified. To date, seven chromosomal loci have been associated with risk for TNBC in multiple studies (Table 1).

## 4. Other Genetic Elements

Genetic elements other than susceptibility genes within the germline may also contribute to risk of TNBC. miRNAs represent a group of nonprotein coding RNAs that alter gene expression by binding to messenger RNA (mRNA) regions and reducing transcription or promoting mRNA degradation. Polymorphisms within the germline may eliminate or create miRNA binding sites or alter the function of miRNAs. For example, patients carrying the A allele for SNP rs743554, located within a predicted miRNA binding site of the integrin beta 4 (*ITGB4*) gene, were found to be at increased risk for ER- breast cancer (OR=2.09; 95% CI=1.19-3.67). Although HER2 status was not included in this study and thus associations with TNBC could not be determined, the A allele was also associated with decreased survival [hazard ration (HR)=2.21, 95% CI=1.21-3.68] [62]. A GWAS analysis of miRNA-associated SNPs performed in women of African ancestry found two SNPs that were associated with increased risk of ER- breast cancer: mir-4725 (rs73991220; OR=1.27, 95% CI=1.09-1.48) and* PAPD4* (rs146287903; OR=0.49, 95% CI=0.33-0.72) [63]. Given the increased risk of TNBC in women of African ancestry, future studies are needed to evaluate the potential role of these two SNPs in TNBC etiology. Similarly, the G allele of rs2910164, located within miR146a which may bind to the 3′ untranslated regions of the* BRCA1* and* BRCA2* genes and thus regulate their expression, has been associated with breast cancer risk (OR=1.77; 95% CI=1.40-2.23) [64]. Although risk for developing specific subtypes of breast cancer was not evaluated for this SNP, the link between* BRCA1* dysfunction and TNBC warrant further investigation.

Recent studies suggest that retroviral sequence elements from ancient retroviral infections may contribute to heritable TNBC. Some members of the human endogenous retrovirus HERV-K family are related to the endogenous mouse mammary tumor virus (MMTV) which can function as a mammary carcinogen in mice. While HML-2 proviruses have not been found at significantly higher frequencies in the genomes of patients with breast cancer compared to healthy controls and have not been associated with breast cancer histology [65, 66], the frequencies of detection for HERV-K113 and HERV-K115 are significantly higher in individuals of African ancestry (21.8% and 34.1%, respectively) compared to individuals from the United Kingdom (4.2% and 1.0%) [67]. Given the enrichment of the TNBC subtype in women of African ancestry, the presence of these sequences should be evaluated in larger populations with available ER, PR, and HER2 status. More recently, Marchi et al. mined whole genome sequence data generated by next-generation sequencing and identified 17 sites of viral integration not present in the human reference sequence [68] that may contribute to breast cancer risk.

Sequences not present in the human reference genome may harbor additional genes and/or genetic elements that contribute to risk of TNBC.* De novo* assembly of whole genome sequences from Asian and African individuals revealed ~5 Mb of novel sequences from both individuals and populations [69]. The authors estimate that a complete human pan-genome would contain 19-40 Mb of novel sequence not present in the current reference genome. Sixty-nine genes, any of which may increase risk of TNBC, were located within unmapped regions of the African genome. Data from our own laboratory demonstrated that the insertion frequency of a 30 Kb region of chromosome 7p11 that harbors the promoter and first three of the four exons that compromise the phophoserine phosphatase-like (*PSPHL) *gene was 76% in African American women compared to only 21% in European American women [70]. While presence of an intact* PSPHL* gene was not associated with increased risk of breast cancer or TNBC, other uncharacterized genes from regions variably represented between populations may contribute to increased risk of TNBC

## 5. Conclusions

Identification of the* BRCA1* gene 25 years ago revolutionized the field of cancer risk assessment. Associations between germline* BRCA1* mutations and TNBC led the National Comprehensive Cancer Network to amend their* BRCA1/2* testing criteria in 2011 to include individuals diagnosed with TNBC [71]. Current guidelines allow testing for patients diagnosed with TNBC at ≤60 years of age with or without a significant family history of breast cancer [72].* BRCA1* and* BRCA2* testing in 439 women with TNBC from the Australian Breast Cancer Tissue Bank supports TNBC pathology as a sufficient criterion for testing as 59% of women with pathogenic mutations did not have a family history of breast or ovarian cancer [34]. In a recent study of 10,901 TNBC patients, pathogenic variants were detected in TNBC risk-associated genes in 4.3% of patients not meeting NCCN testing criteria (diagnosed at >60 years of age without a family history) [35]. Because many of the mutations detected in this group were clinically actionable, the authors suggest that all patients with TNBC may benefit from genetic testing.

The importance of identifying the genetic etiology of TNBC extends beyond risk assessment. Surgical options are not dictated by tumor subtype; however,* BRCA1* and* BRCA2* mutation carriers are at increased risk for contralateral breast and ovarian cancer; thus the option of mastectomy with or without prophylactic removal of the contralateral breast and salpigo-oophorectomy should be considered. For carriers of mutations in other TNBC genes, such as* BARD1* and* PALB2*, evidence is not yet sufficient to recommend mastectomy or oophorectomy [72]. Because patients with TNBC do not respond to hormone- or HER2-targeted treatments, chemotherapy is the primary treatment option. Carcinomas from patients with TNBC from patients with pathogenic* BRCA1/BRCA2* mutations have demonstrated unique sensitivity to platinum agents in both the neoadjuvant and adjuvant settings [73]. More recently, PARP inhibitors, which exploit DNA repair deficiencies in cells with dysfunctional BRCA1 or BRCA2 proteins, leading to synthetic lethality, have shown promise with Olaparib approved by the FDA for the treatment of TNBC in patients with germline* BRCA1/BRCA2* mutations [74].

Despite recent achievements in identifying additional TNBC susceptibility genes and optimizing patient management for mutation carriers, future studies are needed. For example, what is the clinical utility of the low-penetrance genes/loci SNPs? In perhaps the largest GWAS to date, comprised of 94,075 cases and 75,017 controls of European ancestry that were derived from 69 different studies, 313 SNPs were assembled into a polygenic risk score (PRS) for both ER positive and ER negative tumors [75]. Preliminary studies evaluating the utility of the PRS are underway in Canada and Europe; however, given that the models were developed using cases and controls of European ancestry, the ability of this assay to accurately determine risk of TNBC in patients of other ancestries, especially African with its higher frequency of TNBC in young women, may be suboptimal. In conjunction, additional studies of nontraditional elements of the genome including retrotransposons and pseudogenes may reveal additional heritable risk factors for TNBC. Finally, for the ~4% of TNBC patients with germline mutations in genes other than* BRCA1* and* BRCA2* [35, 41], effective management strategies and novel therapeutics are urgently needed.

## Figures and Tables

**Table 1:** Odds ratios for TNBC for loci identified in more than one study.

Location	Gene	SNP	Risk Allele	OR/HR	95% CI	P-value	Reference
1q32	*MDM4*	rs4245739	C	1.18	1.13-1.23	4.3x10^−15^	[15]

			C	1.17	1.09-1.26	3.1x10^−5^	[16]

			C	1.19	1.11-1.29	4.0x10^−6^	[17]

2q35		rs13387042	G	0.93	0.87-1.00	0.049	[17]

			G	1.12	1.05-1.21	0.001	[18]

5p15	*TERT*	rs10069690	A	1.28	1.23-1.33	2.4x10^−33^	[15]

			A	1.24	1.14-1.34	1.4x10^−7^	[17]

			A	1.25	1.16-1.34	1.1x10^−9^	[19]

			A	1.27	1.20-1.36	5.2x10^−14^	[20]

		rs2736108	T	0.77	0.69-0.87	8.3x10^−6^	[17]

6q25	*ESR1*	rs2046210	A	1.16	1.08-1.24	5.3x10^−5^	[17]

			A	1.23	1.16-1.31	5.5x10^−12^	[20]

			A	1.29	1.17-1.42	4.4x10^−7^	[21]

		rs3757318	A	1.33	1.17-1.51	9.3x10^−6^	[17]

		rs12662670	G	1.33	1.15-1.53	1.1x10^−4^	[21]

14q24	*RAD51L1*	rs999737	T	0.86	0.80-0.93	3.0x10^−4^	[21]

			T	0.89	0.80-0.98	0.02	[22]

		rs2588809	A	0.91	0.83-1.00	0.041	[17]

16q12	*TOX3*	rs3803662	A	1.09	1.01-1.17	0.022	[17]

			A	1.21	1.11-1.30	3.1x10^−6^	[18]

			A	1.17	1.09-1.26	3.7x10^−5^	[21]

19p13		rs8170	A	1.26	1.16-1.37	1.3x10^−7^	[17]

			T	1.27	1.17-1.38	2.3x10^−8^	[21]

			A	1.28	1.16-1.41	1.2x10^−6^	[23]

			A	1.25	1.18-1.33	3.3x10^−13^	[24]

		rs2363956	C	0.82	0.77-0.88	2.3x10^−8^	[17]

			C	0.80	0.74-0.87	1.1x10^−7^	[23]

		rs8100241	A	0.81	0.76-0.86	2.4x10^−13^	[20]

			A	0.84	0.78-0.90	8.7x10^−7^	[21]
